# Genome-Wide Identification and Expression Profiling of the *SRS* Gene Family in *Melilotus albus* Reveals Functions in Various Stress Conditions

**DOI:** 10.3390/plants11223101

**Published:** 2022-11-15

**Authors:** Biao Ma, Lili Nian, Noor ul Ain, Xuelu Liu, Yingbo Yang, Xiaolin Zhu, Fasih Ullah Haider, Ying Lv, Pengpeng Bai, Xiaoning Zhang, Quanxi Li, Zixuan Mao, Zongyang Xue

**Affiliations:** 1 College of Resources and Environmental Sciences, Gansu Agricultural University, Lanzhou 730070, China; 2 College of Forestry, Gansu Agricultural University, Lanzhou 730070, China; 3 Centre of Genomics and Biotechnology, Fujian Agriculture and Forestry University, Jinshan, Fuzhou 350002, China; 4 College of Agronomy, Gansu Agricultural University, Lanzhou 730070, China; 5 Key Laboratory of Vegetation Restoration and Management of Degraded Ecosystems, South China Botanical Garden, Chinese Academy of Sciences, Guangzhou 510650, China; 6 University of Chinese Academy of Sciences, Beijing 100039, China

**Keywords:** *MaSRS*, gene family, *Melilotus albus*, gene expression analysis

## Abstract

The plant-specific *SHI*-related sequence (*SRS*) family of transcription factors plays a vital role in growth regulation, plant development, phytohormone biosynthesis, and stress response. However, the genome-wide identification and role in the abiotic stress-related functions of the *SRS* gene family were not reported in white sweet clover (*Melilotus albus*). In this study, nine *M. albus SRS* genes (named *MaSRS01*-*MaSRS09*) were identified via a genome-wide search method. All nine genes were located on six out of eight chromosomes in the genome of *M. albus* and duplication analysis indicated eight segmentally duplicated genes in the *MaSRS* family. These *MaSRS* genes were classified into six groups based on their phylogenetic relationships. The gene structure and motif composition results indicated that *MaSRS* members in the same group contained analogous intron/exon and motif organizations. Further, promoter region analysis of *MaSRS* genes uncovered various growth, development, and stress-responsive *cis*-acting elements. Protein interaction networks showed that each gene has both functions of interacting with other genes and members within the family. Moreover, real-time quantitative PCR was also performed to verify the expression patterns of nine *MaSRS* genes in the leaves of *M. albus*. The results showed that nine *MaSRSs* were up- and down-regulated at different time points after various stress treatments, such as salinity, low-temperature, salicylic acid (SA), and methyl jasmonate (MeJA). This is the first systematic study of the *M. albus SRS* gene family, and it can serve as a strong foundation for further elucidation of the stress response and physiological improvement of the growth functions in *M. albus*.

## 1. Introduction

Transcription factors (*trans*-acting elements, Tfs), are protein molecules with specific modular domains performing core functions in transcription by recruiting target DNA and playing an essential role in plant growth and development [1]. *SHI*-related sequence (*SRS*) is a family of plant-specific Tfs, known as short internodes (*SHI*) or *STY/SRS/SHI* family, that comprises two distinct conserved regions [2]. The first region is a ring-shaped zinc finger structure (CX2CX7CX4CX2C2X6C) that contains the C3HC3H motif and is referred to as the RING region [3]. This ring-shaped conserved domain was first discovered in an African clawed frog (*Xenopus laevis*) and identified as a DNA binding motif [4]. The ring domain comprises cysteine-rich residues that couple with zinc atoms [5], bind to RNA, proteins, and lipid substrates and enhance their conservation [6]. This suggests that *SRS* may play an essential role in multicellular physiological and biochemical processes [4]. The second region is a short IXGH domain at the C-terminus, which is required for homologous isomerization and contains transcriptional activators from acidic amino acids [7]. Some members of the *SRS* family have lost this domain during evolution [8,9].

*SRS* TFs play imperative roles in plant organ growth, carpel development, photomorphogenesis, photoperiod regulation, and phytohormone response [10,11]. In *Arabidopsis*, 11 *AtSRS* genes (*AtSRS3*, *AtSRS4*, *AtSRS5*, *AtSRS6*, *AtSRS7*, *AtSRS8*, *AtSHI*, *AtSTY1*, *AtSTY2*, *AtLRP1*, and *AT1G32730*) have been identified and play essential roles in *Arabidopsis* growth and development [12,13]. Overexpression of *AtSRS5* directly activates the expression of *HY5*, *BBX21,* and *BBX22* genes, thereby regulating photomorphogenesis in the dark [14], and can also inhibit the expression of *LBD16* and *LBD29* genes, leading to hindered lateral root formation [15]. The partially redundant *AtSTY1* and *AtSTY2* genes promote pistil development and stigma formation in a dose-dependent manner in *Arabidopsis* [8]. Another study revealed that *SRS* Tfs in barley (*Hordeum vulgare*) could regulate hormone biosynthesis, and inflorescence growth, inhibit gibberellin (GA), induce barley amylase expression and also control the awn elongation of barley, regulate pistil morphology and promote flowering [16,17]. Studies have also shown that *SRS* gene expression patterns and biological functions of *SRS* are quite diverse. For example, only the gene *GRMZM2G077752* was strongly expressed in senescent leaves of maize (*Zea mays*). In contrast, the other ten *SRS* genes were not expressed at all in senescent leaves because *GRMZM2G077752* may be responsive to abscisic acid (ABA) signals and activate carbohydrate reactivation in senescent leaves [18]. This suggests that the interaction between the *SRS* gene and plant hormone-responsive elements may stimulate plant leaf senescence. Five *OsSRS* genes in *japonica* rice (*Oryza sativa* subsp. *Japonica*) indicated different responses to different abiotic stresses and hormone treatments. Interestingly, GA induced the expression of one *SRS* gene, while paclobutrazol (PB) inhibited the expression, suggesting the antagonistic effects of GA and PB [3]. In addition, 21 members of the soybean (*Glycine max*) *SRS* demonstrated similar expression patterns subjected to drought, salinity, and exogenous ABA could induce the expression of these genes. Zhao and his co-workers’ selected drought and salinity treatments for further functional screening of the *GmSRS18* gene. The results showed that *GmSRS18* was a negative regulator in the drought and salt stresses signaling pathway [2]. It was reported that 27 *SRS* genes in alfalfa (*Medicago sativa*) stem tissue were significantly induced by cold and salt stress and exhibited differential expression patterns, showing that *MsSRSs* may play essential roles in stem tissue-dependent regulatory networks [1].

White sweet clover (*Melilotus albus*) is native to temperate regions of Europe and Asia and is cultivated worldwide [19]. The plant has a great cold, drought, and salt tolerance [20]. It is reported that the yield and nitrogen-fixing ability of *M. albus* are better than those of alfalfa. It is vital in improving soil quality and is often regarded as excellent green manure and feed [21,22]. In recent years, many researchers have identified and analyzed *SRS* transcription factors and their functions in plants such as maize (*Zea mays*) [18], *Arabidopsis* (*Arabidopsis thaliana*) [23], barley (*Hordeum vulgare*) [17], rice (*Oryza sativa* Indica) [3], soybean (*Glycine max*) [2], and alfalfa (*Medicago sativa*) [1]. However, the genome-wide identification of the *SRS* family in *M. albus* and the molecular regulatory mechanism under abiotic and abiotic stress have not been reported. In this paper, *MaSRS* was extracted from the published wide genome sequence information of *Melilotus albus* [19]. Therefore, the primary aim of this study was to identify the putative members of the *MaSRS* gene family and explore the subsequent basic physicochemical properties, phylogeny, gene structure, and protein interaction of nine *MaSRS* genes using bioinformatics and computational methods. The relative expression of *MaSRS* family members at different time points under various stress conditions was also investigated using PCR (qRT-PCR) technology. This study serves as a theoretical framework for analyzing *SRS* genes in plants, which will help in understanding the stress response behaviors that might be helpful for future breeding programs.

## 2. Results

### 2.1. Identification and Analysis of SRS Family Members in M. albus

After a series of screening and identification, nine *SRS* genes were identified in *M. albus* and named *MaSRS01-MaSRS09* according to the position of these genes on the chromosome. The amino acid lengths of these genes varied from 197 (*MaSRS08*) to 368 (*MaSRS06*) and, correspondingly, the relative molecular weight ranged from 22.52211 (*MaSRS08*) kDa to 40.82662 (*MaSRS06*) kDa. The average molecular weight was 34.55999 kDa. Isoelectric points of the encoded proteins ranged from 5.79 (*MaSRS07*) to 8.79 (*MaSRS09*), and the instability index ranged from 47.07 (*MaSRS09*) to 61.44 (*MaSRS05*), with a difference of 14.37. The GRAVY values of the *MaSRS* proteins were all less than 0, indicating that these proteins were hydrophilic. The results of subcellular localization showed that all members of the *MaSRS* family were localized in the nucleus (Table 1).

### 2.2. Chromosomal Locations and Gene Duplication Analysis of MaSRS Genes

**Figure 1:**
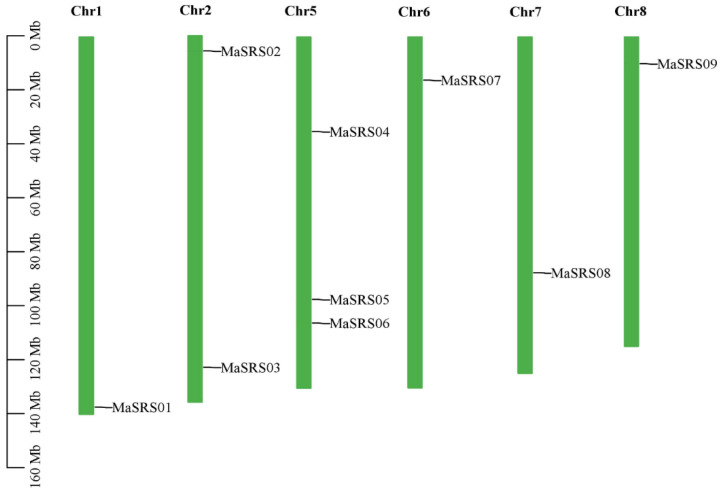
Chromosome distribution of *MaSRS* genes. Note: chromosome number is indicated at the top of each bar. Chromosomal distances are indicated in Mb.

**Figure 2:**
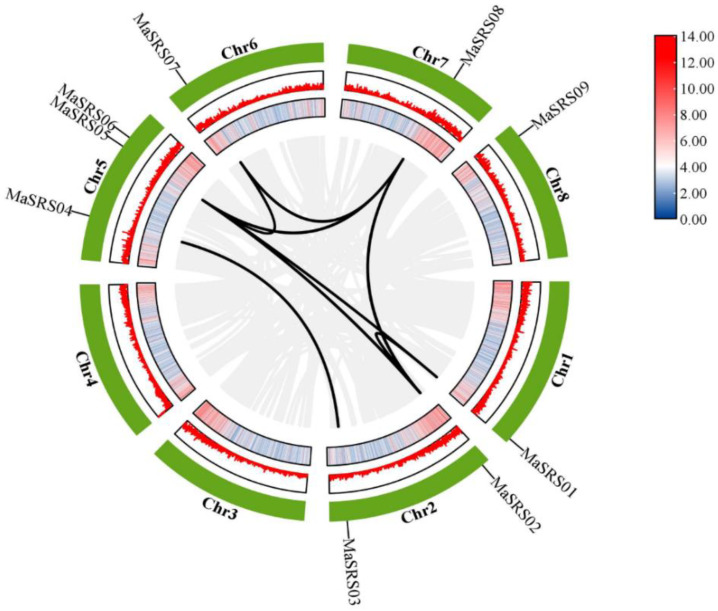
Gene density and synteny analysis of *MaSRS* genes in *M. albus*. Note: the eight chromosomes are shown as green partial circles at the outermost of the large circle, and the gene IDs are shown at the top of each bar. The middle and inner bars are the gene densities as a heatmap and a linear plot, respectively. The background gray lines represent all syntenic blocks of the *M. albus* genome, and the black lines represent duplicated *MaSRS* genes. The data bars color transition from blue to red which indicates that the gene distribution is denser.

The chromosome distribution map showed that nine *MaSRS* members were distributed on six chromosomes (Figure 1), three members on Chr5 (*MaSRS04, MaSRS05, MaSRS06*), two members on Chr2 (*MaSRS02, MaSRS03*), and only one member on Chr1 (*MaSRS01*), Chr6 (*MaSRS07*), Chr7 (*MaSRS08*), Chr8 (*MaSRS09*). Segmental duplications and tandem duplications contribute to the evolution of gene families. According to Figure 2 and Table 2, Duplication analysis indicated eight pairs of segmental duplication: *MaSRS01*, *MaSRS02*; *MaSRS01*, *MaSRS05*; *MaSRS02*, *MaSRS05*; *MaSRS02*, *MaSRS08*; *MaSRS03*, *MaSRS04*; *MaSRS05*, *MaSRS07*; *MaSRS05*, *MaSRS08* and *MaSRS07*, *MaSRS08*. However, no tandem, proximal, or disperse duplicate was found in the *MaSRS* gene family, and thus only segmental duplication seems to have participated in the evolution of the *M. albus SRS* gene family. To investigate potential selective pressure for *MaSRS* gene duplication events, we calculated the nonsynonymous (Ka) and synonymous (Ks) substitution rates. All segmentally duplicated gene pairs in *M. albus* showed Ka/Ks ratios < 1, indicating that they evolved mainly under purifying selection. In *M. albus*, the divergence time of segmental duplication events was about 79 to 360 million years ago (Mya).

### 2.3. Phylogenetic Relationship, Gene Structure, Conserved Motif, and Domain Analysis of MaSRS Family

**Figure 3:**
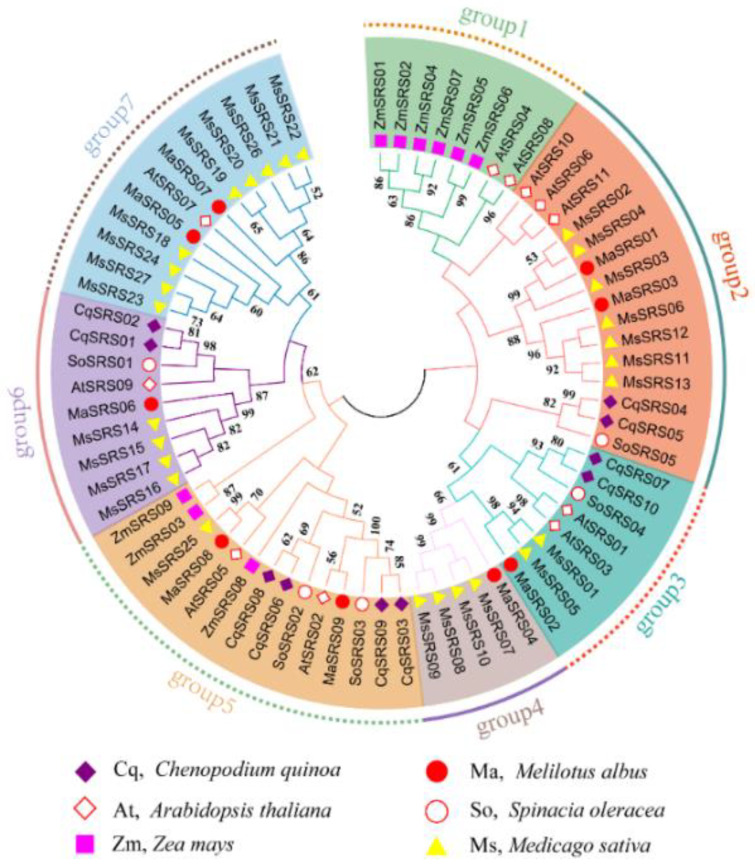
Phylogenetic tree comprising *SRS* genes from quinoa (*Chenopodium quinoa*), Arabidopsis (*Arabidopsis thaliana*), maize (*Zea mays*), white sweet clover (*Melilotus albus*), spinach (*Spinacia oleracea*), and alfalfa (*Medicago sativa*). Note: the phylogenetic tree was constructed using MEGA 7.0 with the maximum-likelihood method with 1000 bootstrap replicates. The tree was classified into seven subgroups, designated group1 to group7. The *CqSRS*, *AtSRS*, *ZmSRS*, *MaSRS*, *SoSRS*, and *MsSRS* proteins were labeled by purple rhombus, red, white rhombus, pink squares, red circles, red-white circles, and yellow triangles, respectively.

To explore the evolutionary relationships between the *MaSRS* family and *SRS* families of other plants, a phylogenetic tree was constructed based on MEGA7.0 software for 71 *SRS* genes in *A. thaliana*, *Z. mays*, *S. oleracea*, *C. quinoa*, *M. sativa*, and *M. albus* (Supplementary Table S1). As shown in Figure 3, the *SRS* gene family members can be classified into seven clusters (no *MaSRS* gene in group 1), which is similar to *AtSRS*. Among them, *MaSRS01* and *MaSRS03* were in group 2, *MaSRS08* and *MaSRS09* were in group 5, and *MaSRS05* and *MaSRS07* were in group 7. The identical clusters of *MaSRS* genes may share a common ancestor and retain similar biological functions during evolution. We found that the closest relationship with the *M. albus SRS* gene family was with alfalfa.

As shown in the phylogeny tree, it is evident that the *SRS* genes of the legume plants (*M. albus* and alfalfa) clustered together. And most of the *M. albus SRSs* also clustered together with proteins from Arabidopsis, quinoa, and spinach, instead of maize, consistent with the closer relationship of *M. albus* to the three dicotyledons. 

**Figure 4:**
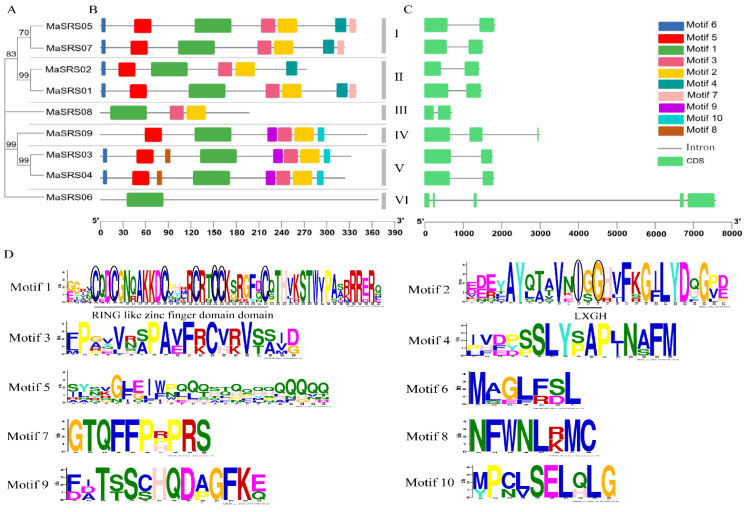
Gene structure and conserved protein domains of *SRS* genes in white sweet clover (*Melilotus albus*) showing exons, introns, and motif sequence organization. (**A**) The unrooted phylogenetic tree of the *MaSRS* family was constructed with 1000 bootstrap replicates, and all *MaSRS* members were classified into six subfamilies. (**B**) Distribution of conserved *MaSRS* domains. (**C**) Intron exon structure of *MaSRS* genes. (**D**) Ten conserved motifs in *MaSRS* proteins.

To better understand the structure and function of the *MaSRS* family, the conserved domains and motifs of nine genes were analyzed (Figure 4). As shown in the figures (Figure 4A,B,D), these genes were classified into six subfamilies (subfamily I, subfamily II, subfamily III, subfamily IV, subfamily V, and subfamily VI) based on the evolutionary tree and conserved motifs, with a similar distribution of conserved motifs in the same subfamily. Motif1 (including the RING domain) is the basic sequence of the *MaSRS* family, which is ubiquitous and most conserved in all genes. Motif2 (with an IXGH structural domain), along with Motif3 and Motif5, are present in eight *MaSRS* genes (except *MaSRS06*) and are relatively well conserved. Motif6 has the least width and is present in six *MaSRSs*. The distribution of Motif3 and Motif4 in *MaSRS* genes was very similar, and it is worth noting that there is only one Motif (Motif1) in *MaSRS06*. These conserved motifs may play an important role in the evolution of the *SRS* gene in *M. albus*.

The distribution of the number and length of introns and exons shows the gene structure of *MaSRS* family members, which showed a relatively simple configuration (Figure 4C). Seventy-eight percent of the *MaSRS* genes had only one intron, *MaSRS09* contained two introns, and *MaSRS06* showed a distinct configuration, containing four introns. The intron sequences of the different genes varied greatly in length, with the intron of *MaSRS06* being the longest, that of *MaSRS09* the second longest, and that of *MaSRS08* the shortest. However, genes with close relatives (*MaSRS01* and *MaSRS02*, *MaSRS03*, and *MaSRS04, MaSRS05*, and *MaSRS07*) had similar exon and intron lengths. Therefore, we speculated that these genes with similar structural features might play similar functions in response to external stimuli in plants.

### 2.4. Identification and Analysis of MaSRS Promoter Cis-Acting Elements

**Figure 5:**
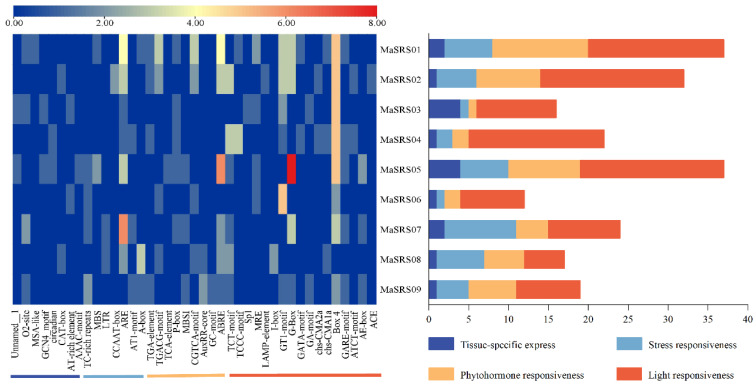
Heat map expression in the promoter region of each *MaSRS* gene and analysis of the number of *cis*-acting elements containing the stress response.

The results of the *cis*-acting elements in the promoter region of the *MaSRS* family showed that this family contained a total of 41 specific *cis*-regulatory elements related to light response [17], plant hormone [9], tissue-specific expression [8], and stress response [7], respectively. The elements for the light response were the most represented, accounting for 41% of the total, while the stress response was the least represented, accounting for only 17% (Figure 5). These specific elements had their unique sequences and performed particular functions that played important roles in regulating gene expression. The gradual transition of color from blue to red in the figure shows that the abundance of the specific elements in the *MaSRS* gene gradually increased. The results show that the action element G-box (CACGTG), which contains six base palindrome sequences, was the most abundant in *MaSRS05*, followed by ABRE (AACCCGG/CACGTG/ACGTG/GCCGCGTGGC). Box4 (ATTAAT) appeared with equal frequency in *MaSRS01-MaSRS05*. Except for *MaSRS06* and *MaSRS09*, the other *MaSRS* genes contained the cis-regulatory element ARE (AAACCA) required for anaerobic induction. The six *MaSRS* genes had a GT1-motif (GGTTAA/GGTTAAT), and individual genes contained some LTR (CCGAAA), TC-rich repeats (GTTTTCTTAC), MBS (CAACTG), GCN4_motif (TGAGTCA), CGTCA-motif (CGTCA), and GA-motif (ATAGATAA), which are related to low-temperature response, defense, drought resistance, involvement in endosperm expression, signaling of the hormone methyl jasmonate (MeJA) and the light response, respectively. 

Different elements in each gene indicate different contributions to the family. *MaSRS01* and *MaSRS05* contained the most *cis*-acting elements, indicating that their functions were complex and diverse, whereas *MaSRS06* was relatively simple. Compared with the other seven *MaSRS* genes, *MaSRS03*, and *MaSRS05* had relatively many tissue-specific expression elements, suggesting that these two genes play important roles in organ development in *M. albus*. *MaSRS07* exhibited the most elements related to stress response, suggesting that it may play an important role in abiotic stress.

### 2.5. Protein Interaction Network Construction and Structure Prediction of MaSRS Tfs

The STRING database contains information about direct physical interactions and potential functions of proteins in several species. Because the protein information of *M. albus* was missing from the database, a *MaSRS* protein interaction network map was constructed based on the protein information of *Arabidopsis* (Supplementary Figure S1). We predicted that seven *AtSRSs* (*AtSRS3*, *AtSRS6*, *AtSRS7*, *LRP1*, *SHI*, *STY1*, and *AT1G32730*) had sequence proximity and thus relates physical interaction and associated functional relationships with *MaSRS* genes, and five *AtSRSs* (*AtSRS4*, *AtSRS8*, *YUC4*, *NGA3*, and *AT3G06840*) had potential interaction with *MaSRSs*. It is worth noting that both *MaSRS06* and *LRP1* contained a conserved domain of Put_zinc_*LRP1*, and *MaSRS06* could act on *YUC4* through *LRP1*. Studies had shown that *YUC4* is an enzyme that activates plant auxin biosynthesis and plays an important role in the growth of *Arabidopsis* seedlings, which could regulate flower shape [24]. In addition, the results showed that proteins (*SHI* and *STY1*) interact with proteins (*YUC4* and *NGA3*). Studies had shown that *NGA3* and *STY3* can induce ovarian transformation into stylar tissue, and also mediate *YUCCA* to promote auxin synthesis in the apical pistil domain [25].

The secondary structure of the *SRS* proteins in *M. albus* was also predicted in this study. The results showed that the nine proteins are mainly composed of the random coil, which accounts for 66.91% on average. The proportion of alpha helix in *MaSRS06* (44.29%) was much larger than in the other *MaSRSs*, while the proportion of extended chain (1.90%) and beta-turn (0.54%) was the smallest (Supplementary Table S2)

**Figure 6:**
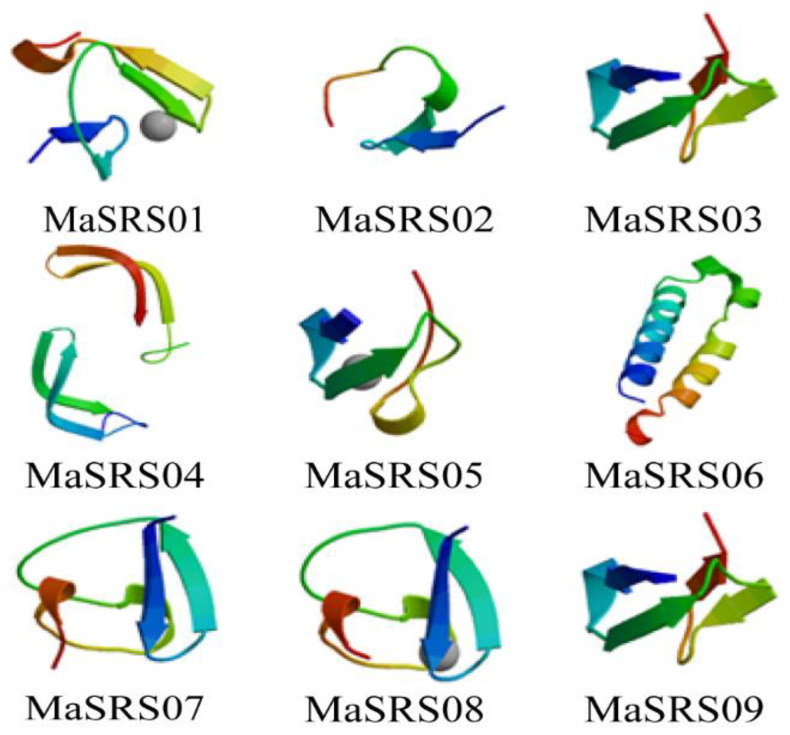
3D structure prediction of *SRS* protein in white sweet clover (*Melilotus albus*).

The prediction results of the 3D structure indicated that homologous proteins genes (MaSRS03 and MaSRS09) had similar 3D structures, but there were also exceptions, such as the difference between *MaSRS01* and *MaSRS02* (Figure 6).

### 2.6. qRT-PCR Analysis of MaSRSs under Different Biotic and Abiotic Stress

**Figure 7:**
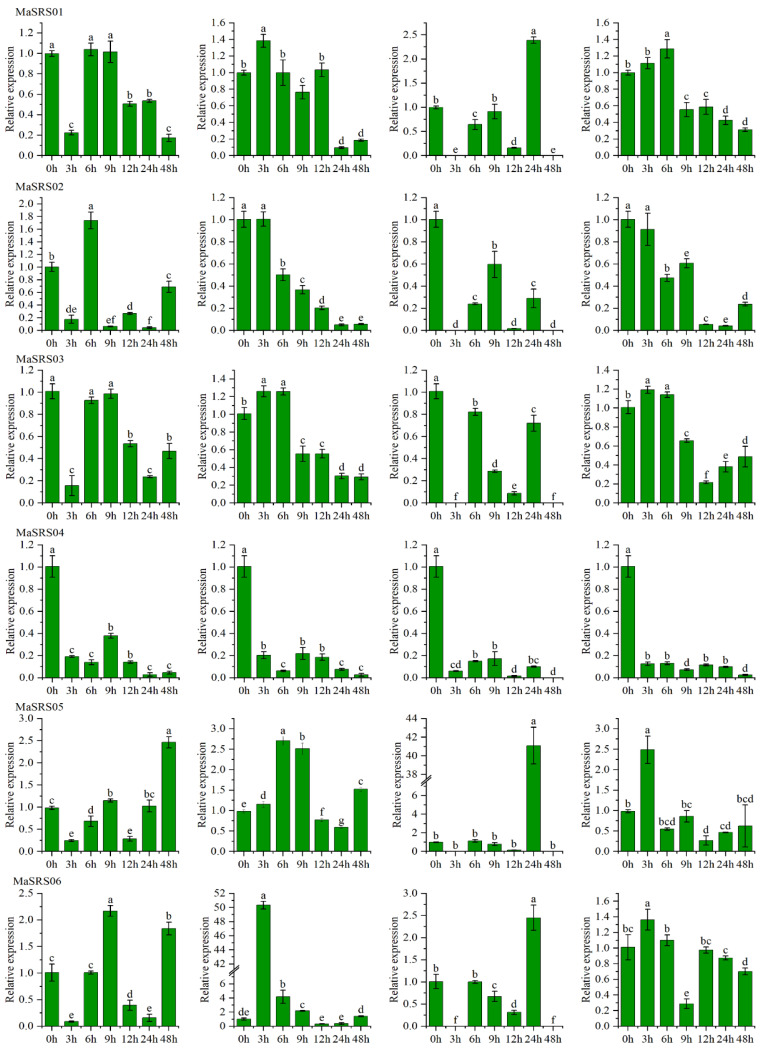

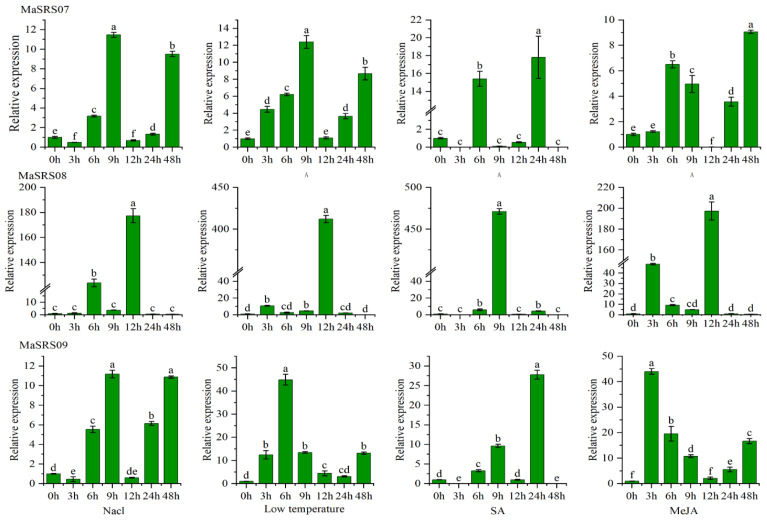
Expression levels of nine *SRS* genes in leaves of white sweet clover (*Melilotus albus*) under salinity, low temperature, salicylic acid (SA) and methyl jasmonate (MeJA) treatments. Note: data represents the means of three independent replicates ± standard deviation (SD). Small letters above the column indicate significant differences in each processing period (a = 0.05, LSD). LSD, least significant difference.

Plant *SRS* genes play an important role in biotic and abiotic stresses. Therefore, the expression patterns of nine *SRS* genes in the leaves of *M. albus* under salinity, low-temperature, SA, and MeJA treatments were investigated in this paper (Supplementary Table S3). The results showed that these genes responded to the four treatments, although the intensity of the response was different. Compared to CK, these genes were up-down-regulated (Figure 7). Among the four treatments, the *MaSRS09* was most significantly up-regulated, indicating high expression, especially under the low-temperature treatment. However, *MaSRS04* showed a consistent down-regulation pattern within 48 h after the four treatments. *MaSRS08* showed a high expression level after 12 h of three treatments (salinity, low-temperature, and MeJA).

Within 3 h after salinity stress, the other eight *MaSRS* genes were down-regulated, except for *MaSRS08*. After 9 h, *MaSRS01*, *MaSRS05*, *MaSRS06*, *MaSRS07*, *MaSRS08*, and *MaSRS09* were up-regulated. After 48 h of treatment, *MaSRS05*, *MaSRS06*, *MaSRS07*, and *MaSRS09* were significantly up-regulated. The expression of *MaSRS* genes at the 6 h and 24 h stress time points were up-regulated and down-regulated, respectively. For example, *MaSRS09* was significantly up-regulated at the 6 h and 24 h stress time points. Within 48 h of cold treatment, the expression levels of *MaSRS07* and *MaSRS09* were significantly up-regulated, whereas the expression levels of *MaSRS02* and *MaSRS04* were down-regulated. The expression levels of *MaSRS05*, *MaSRS06*, *MaSRS07*, *MaSRS08*, and *MaSRS09* were high during the 9 h treatment. Within the 3 h treatment, the expression of *MaSRS02* was not significant, *MaSRS04* was significantly down-regulated, and other *MaSRSs* were significantly up-regulated. Interestingly, the expression of *MaSRS06* was highest 3 h after treatment, approximately ten times higher than at other time points. The relative expression levels of *MaSRS02*, *MaSRS03*, and *MaSRS04* exposed to SA for a prolonged period (within 48 h) were down-regulated. Five *MaSRSs* (*MaSRS05, MaSRS06, MaSRS07, MaSRS08*, and *MaSRS09*) exhibited high expression levels within 24 h, which was consistent with the trend of expression levels within 9 h under cold stress, suggesting that these genes were more sensitive to low temperatures. It is worth noting that *MaSRS05, MaSRS07*, and *MaSRS09* had the highest expression levels within 24 h, which was a dozen times higher than other time periods. The expression level of *MaSRS08* was particularly high at 9 h, exceeding 470, several hundred times higher than at other time points. More interestingly, nine *MaSRSs* were barely expressed at 3 and 48 h. The expression of *MaSRS07* and *MaSRS09* was higher than that of CK within 48 h after MeJA stress. The expressions of six *MaSRSs* (*MaSRS01*–*MaSRS06*) were down-regulated at 9, 12, 24, and 48 h after treatment, and the expression of six *MaSRSs* (*MaSRS01*, *MaSRS02*, *MaSRS03*, *MaSRS06*, *MaSRS07*, and *MaSRS09*) was up-regulated at 6 h after treatment. It is noteworthy that *MaSRS08* and *MaSRS09* were highly expressed after 3 h of treatment, and their expression levels were about 20 and 45, respectively. Among them, *MaSRS08* had the highest expression level at 12 h, which was a dozen times higher than at other time points.

## 3. Discussion

In the first half of the 20th century, *M. albus* was considered the king of green manure and legume forage in the southern and midwestern United States [26], exhibiting the biological characteristics of cold and drought tolerance and adaptation to growth in alkaline soils. Several studies have shown that the *SRS* family is involved in various complex regulatory processes in growth and development [27,28]. However, the *SRS* family has been extensively studied in various plants, including *A. thaliana* [29,30], *Z. mays* [18], *H. vulgare* [16], *O. sativa subsp. Japonica* [31], *P. vulgaris* [4], *M. sativa* [1]. However, in *M. albus*, the *SRS* family has not yet been reported. Therefore, the current study performed a genome-wide analysis of the *MaSRS* family.

In this study, nine *MaSRS* genes were identified and classified into six subfamilies (subfamily I, subfamily II, subfamily III, subfamily IV, subfamily V, and subfamily VI) (Figure 3). The *SRS* genes are generally rare in plants. Twenty-seven *MsSRSs* have been identified in alfalfa, classified into seven subfamilies. The number of *MsSRS* is much higher than that of *A. thaliana*, *Z. mays*, *S. oleracea*, *C. quinoa*, *M. sativa,* and *M. albus*. The main reason may be related to the tetraploid genome and the complex evolution of alfalfa [32]. Studies demonstrated that gene duplication plays a vital role in the expansion of gene families and contributes to the diversification of biological functions [33]. For example, twenty-nine pairs of duplicated genes were found in the *MsSRS* family, including two tandem repeats and twenty-seven segmental repeats [1]. In this study, segmental duplication gene pairs were located on different chromosomes, and no tandem duplication occurred, indicating that the *MaSRS* family expansion was dominated by segmental duplication (Figure 2).

The results of subcellular localization showed that all *MaSRSs* are localized in the nucleus and were mainly involved in regulating gene transcription. The number of amino acids, PI, MW, and instability index of *MaSRS* family members was different (Table 1). For example, the PI value of *MaSRS09* was the largest, and the instability index was the smallest, which was probably closely related to the diversity of *MaSRS* structure and biological function. We also found that closely related genes in the same subfamily share similar motif structures (Figure 4); for example, *MaSRS03* and *MaSRS04* contain eight identical motifs and one intron. Therefore, the composition of motifs and exon-intron combinations may affect gene function. Overall, all *MaSRS* genes contained at least one intron, which was consistent with the results of rice *SRS* genes research [3]. However, *MaSRS* comprised different introns and exons, leading to alternative splicing and may show diversified functions. Recently in 2020, an article on maize mentioned that two members of the *ZmSRS* family had no introns [18]. Based on this, it is speculated that gene expression function may be closely related to gene structure. For example, studies by [34] have shown that introns improved transcription mainly by affecting transcription rate, nuclear output, and transcriptional stability.

To better understand the evolutionary relationship of the *MaSRS* family, the phylogeny and evolution of *SRS* genes in *M. albus* and five other plants were analyzed, and 71 genes were grouped into seven clusters according to evolutionary relationships (Figure 3). This was consistent with the evolutionary grouping of alfalfa by [1]. The closely related *MaSRS05* and *MaSRS07* belonged to the same group phylogenetically, indicating that the two genes were conserved in the monoploid genome and were stable in evolution. Remarkably, *MaSRS06* had one motif and four introns that were sharply different from the other gene structures. Therefore, the presumed gene function is unique. To further confirm, this study analyzed the interaction network of *MaSRS* proteins concerning the interaction of *SRS* proteins in the model plant *Arabidopsis* and found that *MaSRS06* has similar functions to *AT1G32730* and can directly interact with other *SRSs* (Supplementary Figure S1.). Unfortunately, the specific biological processes of *AT1G32730* remain unclear. Thus, variations in gene structure might result in altered functions of *MaSRS06* which needs to be verified by functional characterization by conducting more experiments. The protein-protein interaction network also suggests that *YUC4* can activate plant auxin synthesis and directly interacts with *Arabidopsis SRS* members (*AtSRS3*, *AtSRS6*, *AtSRS7*, *AtLRP1*, *AtSHI*, *AtSTY1*) to promote seedling growth and regulate flower shape [9].

In this study, the secondary structure of the *MaSRS* protein was analyzed, and the 3D structure was predicted (Figure 6 and Supplementary Table S2). The results showed that all *MaSRSs* secondary structures are mainly random coils and do not involve the conformation of amino acid residues. It is well known that hydrogen bonds are destroyed during the folding of some amino acids and that the energy contained in the conformation of side chains conflicts with the maximal hydrogen bonds [35]. The 3D structure prediction also showed that the 3D structures of homologous proteins were similar. The 3D gain of information from the evolutionary process. This is consistent with the research findings of [36].

Over time, plants have evolved a variety of ways to adapt to the environment to survive. Gene expression has been the primary way for plants to address biotic and abiotic stresses. However, the expression of genes was regulated by their promoters or Tfs. For example, the *cis*-acting elements in the *MaSRS* promoter region could reflect the role of family members in biological and abiotic stress responses (Figure 5). Therefore, it was essential to analyze the *cis*-acting elements of the promoters to investigate the functions of specific genes [37,38]. These *MaSRSs* contained different types and numbers of promoter elements, suggesting that each gene might have its unique expression function. For example, except for *MaSRS06* and *MaSRS09*, the other *MaSRSs* contained the cis-regulatory element ARE, which was necessary for anaerobic induction, indicating that ARE is highly conserved in *MaSRS* and may regulate stress expression of environmental adaptation genes. Six *MaSRS* genes contained ABRE, which may be related to salinity stress tolerance in plants [39,40]. The experimental results showed that the combination of ABRE-binding protein *OsBZ8* and ABRE elements could mediate the salinity stress tolerance of rice (*Oryza sativa* Indica) [41]. Therefore, *M. albus* leaves were subjected to salinity stress, and it was found that nine *MaSRSs* were expressed at different time points after treatment. The expression levels of *MaSRS08* and *MaSRS09* were relatively high. Analysis of the *SRS* promoter region revealed that all *MaSRSs* had light-responsive elements (Box4, G-box, or Ga-Motif, etc.). The incident light essential factor for plant growth, and the promoter sequences of most members of the gene family contain multiple light-responsive elements. For example, there were numerous light-responsive elements in the promoter regions of *CDPK* and *PYL* [32,42]. Studies have also shown that the *ZmRXO1* promoter can be regulated by light [43]. In addition, the light response element Box 4 was also found to be regulated by MeJA, ethylene, ABA, and other related hormones [44]. 

Previous studies have shown that the *SRS* gene plays an important role in plant organ growth, carpel development, stress, photomorphogenesis, plant hormone response, and induced lateral root formation (Figure 7) [23,45,46]. Lyu and his co-workers found that the specific expression of genes in plant tissues and organelles is closely related to their functions [47]. The q-PCR quantitative results showed that under the four treatments, all *MaSRS* were expressed to varying degrees in leaves. Notably, most *MaSRS* genes were significantly up-regulated after 3 h of low-temperature treatment, most genes were down-regulated after 3 h of salinity stress treatment, and most *MaSRS* genes were down-regulated after 6 h of SA and MeJA treatment. This confirmed that *MaSRS* could regulate cold and salinity stress and plant growth and development. To a certain extent, this explains that *M. albus* grows in cold and drought-tolerant environments, which provides a theoretical basis for the cultivation of *M. albus* in the later stage.

## 4. Materials and Methods

### 4.1. Identification of SRS Genes in Melilotus albus

The genome sequence data of *M. albus* was obtained from Lanzhou University [19]. *SRS* protein sequences of *Arabidopsis thaliana,* maize (*Zea mays*), spinach (*Spinacia oleracea*), quinoa (*Chenopodium quinoa*), and alfalfa (*Medicago sativa*) were downloaded from the PlantTFDB database (http://planttfdb.gao-lab.org/, accessed on 8 March 2022). *M. albus SRS* gene sequences were identified using BLASTP and Hidden Markov Models (HMM). The conserved domains of the identified *SRS* proteins were further confirmed using the online databases, i.e., NCBI-CDD (https://www.ncbi.nlm.nih.gov/cdd/, accessed on 3 April 2022), the Pfam (http://pfam.xfam.org, accessed on 3 April 2022), and SMART (http://smart.embl-heidelberg.de/, accessed on 3 April 2022). ExPASy ProtParam tool was used to analyze the physicochemical properties such as molecular weight (MV), theoretical isoelectric point (PI), hydrophilicity (GRAVY), number of amino acids, and stability index of *SRS* protein in *M. albus* (https://web.expasy.org/protparam/, accessed on 26 April 2022) [48]. In addition, the Psort-Prediction website was used to determine subcellular localization (http://psort1.hgc.jp/form.html, accessed on 4 May 2022) [49].

### 4.2. Analysis of Synteny, Gene Duplication, and Phylogenetic

The general feature format file (GFF3) annotation file was used in the TBtools tool to draw the distribution map of *SRS* family members of *M. albus* on chromosomes. The MCScanX function in TBtools (Version: 0.6656, Creator: Chengjie Chen, Software source: Guangzhou, Guangdong, China) [50] was used to identify and analyze syntenic blocks of the *MaSRS* genes. The circos program in TBtools (http://circos.ca, accessed on 6 July 2022) was used to generate synteny analysis and chromosomal location diagrams. Non-synonymous (Ka) and synonymous (Ks) rates of *MaSRS* gene duplication pairs were calculated with TBtools. The Ks values were used to calculate the divergence time with the following formula: T = Ks/2λ (λ = 6.56 × 10^−9^) for *M. albus.* Multiple sequence alignments of the amino acid sequences of *A. thaliana*, *Z. mays*, *S. oleracea*, *C. quinoa*, *M. sativa,* and *M. albus* were used in the ClustalW tool. Based on the maximum likelihood method (ML) of the MEGA 7.0 software, the phylogenetic tree was constructed [51], using JTT + G as the model using the bootstrap verification parameter to 1000. Finally, the constructed tree file was uploaded to Adobe Illustrator 2021 (AI) to beautify and better display it.

### 4.3. Analysis of the Conserved Motifs and Gene Structures

The genomic data were imported into GSDS online software (http://gsds.cbi.pku.edu.cn/, accessed on 3 April 2022) in GFF3 annotation file format to analyze the structural features of the genes [52] and map the genetic structure using TBtools [50]. The analysis of the conserved motifs of amino acids was performed using the MEME Suite (Motif-based sequence analysis tools) (MEME 4.11.1; https://meme-suite.org/, accessed on 28 March 2022) online software [52,53], setting the motif number to ten and the other parameters to the default values.

### 4.4. Analysis of Cis-Acting Elements of MaSRS Genes, MaSRSs Protein-Protein Interaction Networks, and Prediction of Protein 3D Structure

Using the information in the GFF3 annotation of the *M. albus* genome, 2000 bp sequences upstream of the transcription start site of each *SRS* gene were extracted using TBtools [50]. Then, *cis*-acting elements in the promoter region of the *SRS* gene were predicted using the PlantCARE website (http://bioinformatics.psb.ugent.be/webtools/plantcare/html/, accessed on 22 April 2022) [54]. Search results were collected, filtered, and presented in a graphical format. Using *A. thaliana* as a template, the *SRS* protein interaction network (confidence > 0.8) was constructed using the STRING database (http://string-db.org, accessed on 27 April 2022), the software [55]. The secondary structure of the protein was analyzed by the SOPM (https://npsa-prabi.ibcp.fr/cgi-bin/npsa_automat.pl?page=npsa_sopma.html, accessed on 26 April 2022) program [56], and the spatial structure of the protein was 3D modeled using SWISS-MODEL, an online database (https://swissmodel.expasy.org/interactive, accessed on 20 March 2022) [57]. Each gene model was also evaluated by Ramachandran plots to increase its credibility [58].

### 4.5. Plant Cultivation and Stress Treatments

The *M. albus* seeds with uniform shapes and full grains were selected. The seed coat was removed from the seeds and sterilized the seeds with 75% alcohol for 3–5 min, and then rinsed with sterile deionized water (4–5 times), and finally transferred to culture dishes with sterile filter paper. These culture dishes were placed in an incubator with a temperature of 24 °C, an illumination period of 16 h, and relative humidity of 55–60%. After the leaves were fully opened, the seedlings were transplanted into the flowerpot for further cultivation. The 4-week-old *M. albus* plants in the same growth state were subjected to four stress treatments: (1) salicylic acid (SA) stress: plants were given 300 mL water per pot having 200 μmol/L SA concentration (2) Methyl jasmonate (MeJA) stress: plants were given 300 mL water per pot, having 200 μmol/L MeJA concentration; (3) salinity stress: plants were supplied with 300 mL water per pot, having 200 μmol/L NaCl concentration; (4) cold stress: 4 °C treatment. *M. albus* plants were sampled at 0, 3, 6, 9, 12, 24, and 48 h after induction of low temperature, i.e., 4 °C. The tissues sampled for RT-PCR analysis were the leaves of *M. albus* on 27 April 2022. Samples were flash frozen in liquid nitrogen and then stored in a freezer at −80 °C for subsequent quantitative experiments.

### 4.6. RNA Extraction and Real-Time Fluorescence Quantitative PCR Analysis of MaSRS

Total RNA was extracted from leaf samples using the SYBR Green Premix Pro Taq HS Premix Extraction Kit (Accurate Biotechnology, Changsha, Hunan, China), and a Nano-Drop 2000 UV spectrophotometer (Thermo Fisher Scientific, Waltham, MA, USA) was used to determine RNA concentration (Thermo Fisher Scientific, Waltham, MA, USA). Next, we reverse-transcribed the RNA with the M-Mu LV first-strand cDNA synthesis kit to obtain cDNA, and cDNA concentration was measured and diluted to 100 ng/μL, which served as a template for RT -qPCR reaction mixture. The expression of 9 *MaSRS* genes at the CK and four stress factors were measured by quantitative real-time fluorescence PCR (RT -qPCR). Perl Primer 6 software was used to design specific primers for this study (Supplementary Table S4). PCR amplification steps: denaturation at 95 °C for 10 s, denaturation at 95 °C for 5 s, and 40 cycles at 60 °C for 30 s of continuous denaturation. In addition, the relative expression levels of *MaSRS* were calculated using the 2^−ΔΔCt^ method [59]. For RT -qPCR, three biological replicates were used for each treatment, and three technical replicates were used for each reaction to eliminate operational errors.

### 4.7. Statistical Analysis

We used SPSS statistical software for one-way ANOVA to analyze the significance of the relative expression of *MaSRS* genes under multiple stress treatments. The treatment means were compared using the LSD test at a significance level, i.e., *p* ≤ 0.05, and graphs were plotted using Origin 2021 software(Version: 9.95, Creator: OriginLab, Software source: Northampton, MA, USA).

## 5. Conclusions

This study was the first comprehensive identification and analysis of the *SRS* gene family in *M. albus*. The nine *MaSRS* genes are located on six of eight chromosomes and are divided into six subgroups. *MaSRS* genes of the same subgroup have similar structures and motifs. There are four types of response elements in the promoter region, among which light-responsive elements are the most common. *MaSRS06* interacts with other *MaSRS* and finally interacts with *YUC4.* This study focused on the expression analysis response of the *MaSRS* family under the four treatments, and the results showed that all genes were up-regulated or down-regulated to varying degrees under the four treatments. In the future, based on the identification and characteristics of these *MaSRS* genes, further functional annotation of these genes will help to cultivate stress-tolerant varieties of *M. albus*.

## Figures and Tables

**Table 1:** Details of the *SRS* gene family members identified in *M. albus*.

Gene ID	Gene Name	Protein Length (aa)	Molecular Weight (kDa)	Isoelectric Point (PI)	GRAVY	Instability Index	Subcellular Localization
Malbus0105973.1	*MaSRS01*	339	36.69790	7.63	−0.845	60.61	Nucleus
Malbus0200518.1	*MaSRS02*	273	29.96145	8.53	−0.562	59.56	Nucleus
Malbus0205193.1	*MaSRS03*	332	36.18219	7.24	−0.696	48.69	Nucleus
Malbus0501767.1	*MaSRS04*	324	35.59567	7.55	−0.653	56.92	Nucleus
Malbus0503375.1	*MaSRS05*	339	36.94744	6.70	−0.750	61.44	Nucleus
Malbus0503792.1	*MaSRS06*	368	40.82662	5.91	−0.804	48.56	Nucleus.
Malbus0601083.1	*MaSRS07*	323	34.95433	5.79	−0.576	55.77	Nucleus
Malbus0702600.1	*MaSRS08*	197	22.52211	8.70	−0.951	59.98	Nucleus
Malbus0800677.1	*MaSRS09*	353	37.35217	8.79	−0.604	47.07	Nucleus

**Table 2:** Duplicated *MaSRS* genes in *M. albus*.

Gene_1	Gene_2	Ka	Ks	Ka_Ks	Duplication Type	T (Mya.)
*MaSRS01*	*MaSRS02*	0.28247617	0.923572967	0.305851492	Segmental	102.6192185
*MaSRS01*	*MaSRS05*	0.412837564	1.299059979	0.317797153	Segmental	144.3399977
*MaSRS02*	*MaSRS05*	0.372374639	1.280259294	0.290858767	Segmental	142.2510326
*MaSRS02*	*MaSRS08*	0.499505712	1.917012554	0.260564654	Segmental	213.0013949
*MaSRS03*	*MaSRS04*	0.224039169	0.718741807	0.311710223	Segmental	79.8602008
*MaSRS05*	*MaSRS07*	0.277413196	1.061327635	0.261383183	Segmental	117.9252928
*MaSRS05*	*MaSRS08*	0.580204202	2.042528126	0.284061793	Segmental	226.9475696
*MaSRS07*	*MaSRS08*	0.449255747	3.245713835	0.138415082	Segmental	360.6348705

Note: T = Ks/2λ; where λ = 4.5 × 10^−9^.

## Data Availability

Not applicable.

## Supplementary Materials

The following supporting information can be downloaded at: https://www.mdpi.com/article/10.3390/plants11223101/s1, Figure S1: Construction of a *MaSRS* protein interaction network map based on *Arabidopsis* homologous protein interaction information; Table S1: The *SRS* protein sequence information in this study; Table S2: Secondary structure prediction of *MaSRS* protein; Table S3: The original data of expression of 9 genes by RT-qPCR; Table S4: *MaSRS* primer design.
