# Supplementary material for: Genome-Wide Identification and Expression Profiling of the SRS Gene Family in Melilotus albus Reveals Functions in Various Stress Conditions
Source: Plants (Basel). 2022 Nov 15;11(22):3101. doi: 10.3390/plants11223101 (PMC9693462; doi:10.3390/plants11223101)
Supplement: Supplementary file 1 [file plants-11-03101-s001.zip › Supplementary Figure S1.pdf]

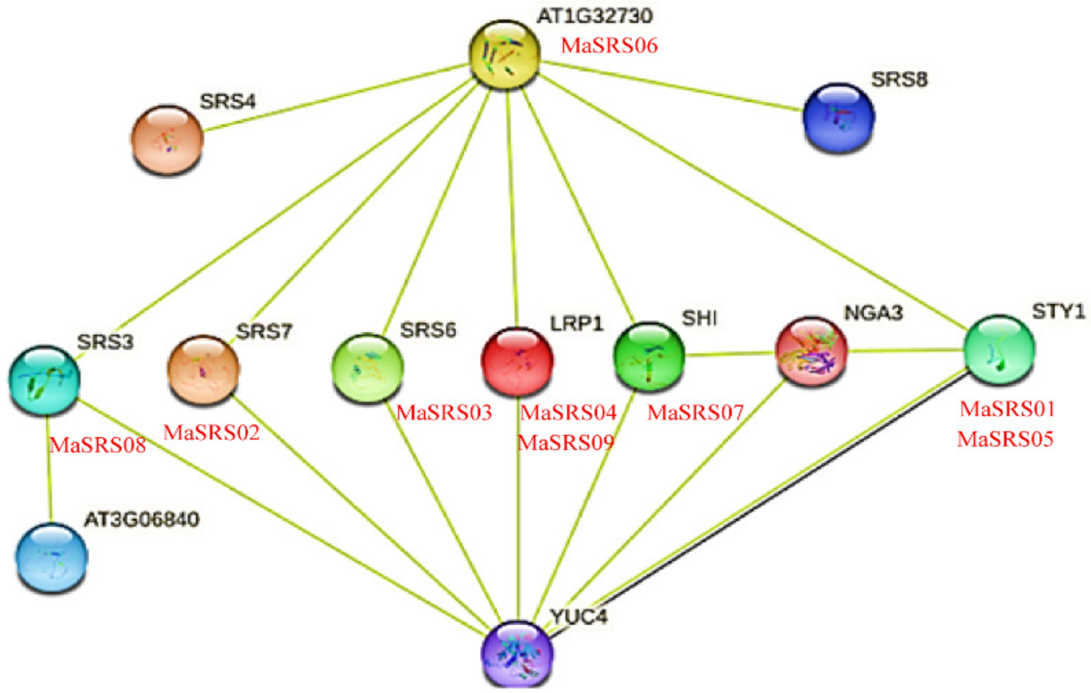

Supplementary Figure S1. Construction of a *MaSRS* protein interaction network map based on *Arabidopsis* homologous protein interaction information
