# Supplementary material for: Genome-Wide Identification and Expression Profiling of the SRS Gene Family in Melilotus albus Reveals Functions in Various Stress Conditions
Source: Plants (Basel). 2022 Nov 15;11(22):3101. doi: 10.3390/plants11223101 (PMC9693462; doi:10.3390/plants11223101)
Supplement: Supplementary file 1 [file plants-11-03101-s001.zip › Supplementary Table S2.pdf]

Supplementary Table S2. Secondary structure prediction of *MaSRS* protein

Gene ID	Gene	Alpha helix (%)	Extended strand (%)	Beta turn (%)	Random coil (%)
Malbus0105973.1	MaSRS01	14.75	14.16	2.65	68.44
Malbus0200518.1	MaSRS02	13.19	13.92	2.93	69.69
Malbus0205193.1	MaSRS03	7.23	16.87	3.31	72.59
Malbus0501767.1	MaSRS04	9.26	17.59	4.32	68.83
Malbus0503375.1	MaSRS05	13.86	12.68	2.95	70.50
Malbus0503792.1	MaSRS06	44.29	1.90	0.54	53.26
Malbus0601083.1	MaSRS07	12.69	14.24	2.79	70.28
Malbus0702600.1	MaSRS08	17.26	16.75	2.54	63.45
Malbus0800677.1	MaSRS09	9.07	18.98	6.80	65.16
Mean value		15.73	14.12	3.20	66.91
